# Editorial: Plant protein termini: Their generation, modification and function

**DOI:** 10.3389/fpls.2022.1040392

**Published:** 2022-09-30

**Authors:** Patrick Willems, Pitter F. Huesgen, Iris Finkemeier, Emmanuelle Graciet, Thierry Meinnel, Frank Van Breusegem

**Affiliations:** ^1^ Department of Plant Biotechnology and Bioinformatics, Ghent University, Ghent, Belgium; ^2^ Vlaams Instituut voor Biotechnologie (VIB) Center for Plant Systems Biology, Vlaams Instituut voor Biotechnologie (VIB), Ghent, Belgium; ^3^ Department of Biomolecular Medicine, Ghent University, Ghent, Belgium; ^4^ Vlaams Instituut voor Biotechnologie (VIB) Center for Medical Biotechnology, Vlaams Instituut voor Biotechnologie (VIB), Ghent, Belgium; ^5^ Central Institute for Engineering, Electronics and Analytics, ZEA-3, Forschungszentrum Jülich, Jülich, Germany; ^6^ Cologne Excellence Cluster on Stress Responses in Ageing-Associated Diseases, CECAD, Medical Faculty and University Hospital, University of Cologne, Cologne, Germany; ^7^ Institute of Biochemistry, Department for Chemistry, University of Cologne, Cologne, Germany; ^8^ Plant Physiology, Institute of Plant Biology and Biotechnology, University of Muenster, Münster, Germany; ^9^ Department of Biology, Maynooth University, Maynooth, Ireland; ^10^ Kathleen Lonsdale Institute for Human Health Research, Maynooth University, Maynooth, Ireland; ^11^ Université Paris-Saclay, Commissariat à l'énergie atomique et aux énergies alternatives (CEA), Centre National de la Recherche Scientifique (CNRS), Institute for Integrative Biology of the Cell (I2BC), Gif-sur-Yvette, France

**Keywords:** protein N-termini, N-terminal modifications, N-degron, Arabidopsis thaliana, alternative splicing, alternative translation initiation

A single gene can give rise to multiple protein species, or proteoforms, by taking advantage of biological processes occurring before, during or after protein synthesis (**Figure 1**). These processes often yield altered protein starts that can influence protein function. For instance, alternative splicing or translation initiation can lead to distinct protein N-termini, while nascent polypeptide chains can be trimmed and N-terminally acetylated during translation. Also, after protein synthesis, protein termini can be recognized by modifying enzymes or by E3 ubiquitin ligases, the latter marking proteins for ubiquitin-dependent degradation by the N-degron pathways. Alternatively, protease processing can generate novel protein fragments (with new N- and C-termini) that can acquire specialized functions. A central method within protein N-terminal biology is positional proteomics, where peptides matching protein N-termini can be enriched and identified, thereby informing on the identity, modification status and intensity of protein N-termini within cells. N-terminal proteomics can be used to delineate alternative proteoforms (e.g. shaped by alternative splicing or translation initiation) or identify substrates of N-terminal modifying enzymes, proteases or N-degron pathways.

**Figure 1:**
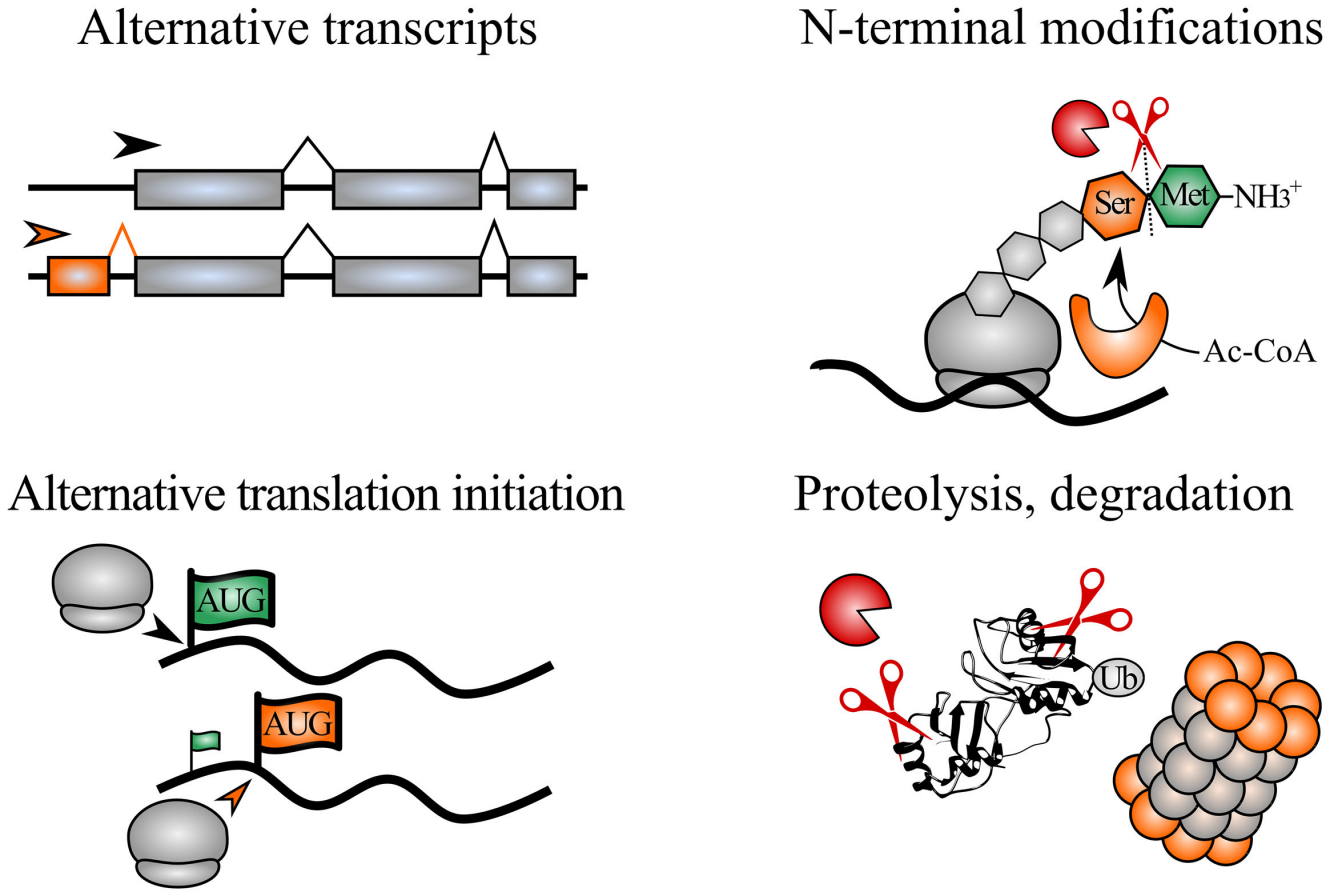
Biological processes shaping distinct N-terminal proteoforms. Expression of alternative transcripts with differing exon-intron structures can encode novel protein N-termini. Alternative translation initiation sites can be selected, thereby shaping truncated and extended N-terminal proteoforms. During translation, initiator Met (green) can be cleaved by Met aminopeptidases, exposing the second residue (here Ser, orange). In addition, protein N-termini can be N-terminally modified, *e.g.* such as co-translational N-terminal acetylation catalyzed by N-terminal acetyltransferases. Lastly, post-translational cleavage of proteins by proteases yields proteolytic fragments that can acquire novel functions or be further degraded, e.g. *via* the ubiquitin proteasome pathway. Ac-CoA, acetyl coenzyme A; Ub, ubiquitin.

In plants, our understanding of the extent and functional implications of protein termini and their corresponding proteoforms is still in its infancy. This Research Topic focuses on the closely intertwined research disciplines studying the generation, modification and function of protein N-termini in plants.

## Alternative splicing and translation initiation

While alternative splicing is a ubiquitous process in plants, their true contribution to proteome complexity remains a debatable topic (Yu et al., 2016). In this Research Topic, Linnenbrügger et al. report on alternatively splicing of glucose-6-phosphate dehydrogenase (G6PDH), which produces N-terminally extended proteoforms, G6PDF5.4 and G6PDF5.5, that localize at the cytosolic side of the endoplasmic reticulum (ER). G6PDH catalyzes the first step of the oxidative pentose-phosphate pathway (OPPP), and OPPP activity at ER subdomains was further supported by the co-localization and co-purification of G6PDF5.4 with downstream OPPP enzymes 6-phosphogluconolactonase PGL2 and 6-phosphogluconate dehydrogenase PGD2. While PGD2 was identified as *S*-palmitoylated (Kumar et al., 2022), ER targeting for PGL2 was shown to be dependent on a C-terminal CaaX prenylation motif. Taken together, these enzymatic OPPP reactions could provide NAPDH for biosynthetic reactions at the cytosolic side of the ER. Evidence for the existence of multiple gene proteoforms can be systematically gained by using OMICS technologies such as N-terminal proteomics and ribosome footprinting (ribo-seq). Here, Willems et al. provide complementary evidence for ~90 proteoform pairs stemming from alternative translation initiation sites (aTIS) in Arabidopsis by combining N-terminal proteomics and translation initiation ribosome footprinting. Next to aTIS mutagenesis validation experiments, N-termini from truncated proteins initiated from aTIS downstream of the canonical AUG start codon resulted were compliant with co-translational modification rules. Genes giving rise to full-length and truncated N-terminal proteoforms often result in alternative targeting of the proteins. For example, the full-length proteins are often targeted to mitochondria and/or chloroplasts, while downstream aTIS sites seem to act as a mechanism to bypass N-terminal targeting peptides and provide cytosolic protein copies.

## N-terminal acetylation and N-degron pathways

Yeast serves as a key model species to study eukaryotic biological systems. In this Research Topic, a novel assay in yeast is described to study the modification and recognition of plant protein N-termini. Meeting the need for a straightforward and more sensitive assay to test and identify putative plant N-degron substrates, Kozlic et al. introduced the Arabidopsis PRT6/N-degron pathway within a *Saccharomyces cerevisiae* strain deficient in its endogenous E3 ubiquitin ligase UBR1, which is also termed N-recognin because of its ability to bind so-called destabilizing N-terminal residues. Co-expression of the Arabidopsis N-recognin PRT6 and ubiquitin conjugating enzyme AtUBC2 successfully reconstituted an active N-degron pathway in yeast. To facilitate high-throughput assays, a GFP fluorescence-based read-out was developed. This confirmed PRT6/N-degron dependent turnover of known substrates in plants, such as HRE2 and ZPR2 (Gibbs et al., 2011; Weits et al., 2019), as well as other tested proteins such as BBX31. Results in yeast differed from *in planta* assays for others, such as the basal immunity regulator RIN4 (Goslin et al., 2019). Also in yeast, the E3 ligase Doa10 was shown to recognize acetylated protein N-termini as N-degron (Ac/N-degrons) and target for ubiquitin-dependent degradation (Hwang et al., 2010). Such Ac/N-degrons can be conditionally exposed in misfolded proteins and functions within ER-associated protein degradation. Another link to ER stress was studied in this Research Topic by Huber et al. in Arabidopsis plants deficient in the NatB complex or in Doa10, which is an ER-membrane resident E3 ubiquitin ligase. The NatB mutants are hypersensitive to salt and osmotic stress (Huber et al., 2020), which both trigger protein misfolding and ER stress. To induce protein misfolding, dithiothreitol (DTT) and tunamycin (TM) were administered to impair structural disulfides and glycoprotein folding, respectively. *NatB* mutants were solely hypersensitive to DTT treatment, excluding a major function for in ER stress. Instead, impaired redox balance likely underlies the observed phenotype, as lower levels of reactive oxygen species were observed and DTT-responsive transcripts were induced in *NatB* mutants. The role of DOA10 in Arabidopsis remains ambiguous, as *doa10* mutants were insensitive to both DTT and TM treatment, ruling out DOA10 as sole N-recognin of Ac/N-degron in plants. To improve the enzymatic characterization of acetyltransferases, Asensio et al. developed a continuous, non-radioactive assay using the *Schizosaccharomyces pombe* SpNatA N-terminal acetyltransferase. Here, the release of coenzyme A (CoA) by NAT activity was coupled to the reaction of pyruvate dehydrogenase that re-generates acetyl-CoA and releases NADH that can be monitored spectrometrically. The assay was first validated using SpNatA, which uncovered a hitherto unknown role for remote basic residues that could interact with a negatively charged region at the extremity of the SpNatA peptide binding site. Showcasing its capacity to characterize new GNAT enzymes, the rice plastidial OsGNAT2 was shown to have a similar activity and substrate specificity as its Arabidopsis orthologue AtGNAT2 (Bienvenut et al., 2020). The assay shows great promise for high-throughput measurements of GNATs, and the identification of their substrates and inhibitors.

## Limited proteolysis

In contrast to protein degradation, protein processing or limited proteolysis can yield protein fragments with acquired functions. In this Research Topic, Heidorn-Czarna et al. review protein processing events within mitochondria in plants, yeast and mammals. In addition to the primary processing of N-terminal presequences, several secondary protein processing events take place in mitochondria. While the action of some proteases is evolutionary conserved, limited proteolysis orchestrated by other mitochondrial proteases such as rhomboid proteases or the ATP23 metalloprotease seem to have evolutionarily diverged. The study of mitochondrial processing events in plants is scarce, necessitating additional studies using N-terminal proteomics to expand the substrate repertoire and study the function of mitochondrial proteases in plants. Staying on the topic of limited proteolysis, De Backer et al. review membrane-bound transcription factors (MB-TFs) that can be released by proteolytic processing or *via* alternative splicing that eliminates the transmembrane domain (TMDs). While a total of 64 high-confidence MB-TFs are present in Arabidopsis (Yao et al., 2017), activation mechanisms are only elucidated for three bZIP TFs. *In silico* analyses pointed out 18 MB-TFs with alternative transcripts lacking TMDs and 11 MB-TFs helix-breaking motifs inside the TMD, which are a typical structural feature facilitating regulated intramembrane proteolysis. For identification of responsible proteases, multiplexed gene editing, N-terminal proteomics, chemical biology, and proximity-based labeling form promising avenues to explore in the future.

## Author contributions

All authors jointly defined the content of the Research Topic and participated in the editing process. All authors listed have made a substantial, direct, and intellectual contribution to the work and approved it for publication.

## Funding

This work was supported by the Research Foundation-Flanders (Junior Postdoctoral fellowship grant no. 12T1722N to PW).

## Acknowledgments

We genuinely acknowledge the contribution of every author, reviewer, and editor that made this Research Topic possible.

## Conflict of interest

The authors declare that the research was conducted in the absence of any commercial or financial relationships that could be construed as a potential conflict of interest.

## Publisher’s note

All claims expressed in this article are solely those of the authors and do not necessarily represent those of their affiliated organizations, or those of the publisher, the editors and the reviewers. Any product that may be evaluated in this article, or claim that may be made by its manufacturer, is not guaranteed or endorsed by the publisher.
